# Hormone-induced protection of mammary tumorigenesis in genetically engineered mouse models

**DOI:** 10.1186/bcr1645

**Published:** 2007-01-26

**Authors:** Lakshmanaswamy Rajkumar, Frances S Kittrell, Raphael C Guzman, Powel H Brown, Satyabrata Nandi, Daniel Medina

**Affiliations:** 1 Department of Pathology, Texas Tech University Health Sciences Center, 4800 Alberta Avenue, El Paso, TX 79905, USA; 2 Department of Molecular and Cellular Biology, Baylor College of Medicine, One Baylor Plaza, Houston, TX 77030, USA; 3 Department of Molecular and Cell Biology and the Cancer Research Laboratory, University of California, Berkeley, 491 Life Science Addition, Berkeley, CA 94720, USA; 4 Breast Center, Baylor College of Medicine, One Baylor Plaza, Houston, TX 77030, USA; 5 Department of Molecular and Cell Biology, University of California, Berkeley, 142 Life Sciences Addition, Berkeley, CA 94720, USA; 6 Department of Molecular and Cellular Biology, Baylor College of Medicine, One Baylor Plaza, Houston, TX 77030, USA

## Abstract

**Introduction:**

The experiments reported here address the question of whether a short-term hormone treatment can prevent mammary tumorigenesis in two different genetically engineered mouse models.

**Methods:**

Two mouse models, the *p53*-null mammary epithelial transplant and the c-*neu *mouse, were exposed to estrogen and progesterone for 2 and 3 weeks, respectively, and followed for development of mammary tumors.

**Results:**

In the *p53*-null mammary transplant model, a 2-week exposure to estrogen and progesterone during the immediate post-pubertal stage (2 to 4 weeks after transplantation) of mammary development decreased mammary tumorigenesis by 70 to 88%. At 45 weeks after transplantation, analysis of whole mounts of the mammary outgrowths demonstrated the presence of premalignant hyperplasias in both control and hormone-treated glands, indicating that the hormone treatment strongly affects the rate of premalignant progression. One possible mechanism for the decrease in mammary tumorigenesis may be an altered proliferation activity as the bromodeoxyuridine labeling index was decreased by 85% in the mammary glands of hormone-treated mice. The same short-term exposure administered to mature mice at a time of premalignant development also decreased mammary tumorigenesis by 60%. A role for stroma and/or systemic mediated changes induced by the short-term hormone (estrogen/progesterone) treatment was demonstrated by an experiment in which the *p53*-null mammary epithelial cells were transplanted into the cleared mammary fat pads of previously treated mice. In such mice, the tumor-producing capabilities of the mammary cells were also decreased by 60% compared with the same cells transplanted into unexposed mice. In the second set of experiments using the activated Her-2/*neu *transgenic mouse model, short-term estradiol or estradiol plus progesterone treatment decreased mammary tumor incidence by 67% and 63%, and tumor multiplicity by 91% and 88%, respectively. The growth rate of tumors arising in the hormone-treated activated Her-2/*neu *mice was significantly lower than tumors arising in non-hormone treated mice.

**Conclusion:**

Because these experiments were performed in model systems that mimic many essential elements of human breast cancer, the results strengthen the rationale for translating this prevention strategy to humans at high risk for developing breast cancer.

## Introduction

It has long been recognized that hormones have an intimate and decisive role in the development and progression of mammary tumorigenesis. The earliest treatment for prevention of breast tumor recurrence in humans (published in 1896) was removal of the ovaries, the source of the reproductive hormones estrogen and progesterone [1]. The most efficacious treatment for the prevention of recurrence in modern therapy for breast cancer is treatment with drugs that target the estrogen pathway, either the estrogen receptor or estrogen metabolism [2]. Many risk factors for breast cancer development involve the hormonal history of the patient, including early age of menarche, late age of menopause, late age of first pregnancy, nulliparity, and estrogen/progesterone hormone replacement exposure. Indirectly, postmenopausal obesity and alcohol consumption are thought to enhance breast cancer risk through altering estrogen levels [3].

Paradoxically, one of the strongest and extensively documented protective factors for breast cancer in both humans and rodent models is short-term stimulation with hormone, either by means of an early full-term pregnancy or with the hormones estrogen and progesterone. The protective properties of estrogen and progesterone were initially shown in 1962 by Charles Huggins [4] and the extensive studies in this area have been reviewed [5,6]. Studies over the past 30 years to document and understand the mechanistic basis for this phenomenon have used the traditional rat and mouse chemical-carcinogen-induced mammary tumor models. These models use etiological agents that are thought not to be risk factors in human breast cancer. One of the known risk factors in human breast cancer is exposure to radiation [7]. Ironically, in the only study done in the rat where radiation was the initiating agent, hormones were not protective [8]. These considerations raise the question of the relevance of the traditional models for understanding the mechanism of the protective effects of hormones.

Over the past 10 years, there have been many new mouse models of mammary cancer based on the enhanced or deleted expression of specific genes known to have a role in human breast cancer [9]. One of the best-studied models is deletion of the *p53 *gene in BALB/c mammary epithelium [10,11]. In this model, the deletion of *p53 *gene expression does not perturb the normal growth and differentiation of the mammary epithelium nor its normal dependence on hormones. However, risk for spontaneous mammary cancer is increased over a 14-month period, and this risk is enhanced by prolonged stimulation with estrogen and progesterone, by irradiation, or by chemical carcinogens [10-12]. The cancers that arise in these mice are locally invasive, exhibit metastases to the lung, and about 20% retain hormone dependence. In addition, the premalignant phenotype mimics that of human ductal carcinoma *in situ *both morphologically and with respect to retaining estrogen receptor expression [11]. A study of gene expression profiles of tumors arising in the *p53*-null mammary epithelium with a randomly selected group of stage 1 and 2 human breast cancers found a large number of genes that were commonly expressed in both sets of tumors [13].

A second and more tumorigenic model is the activated Her-2/*neu *(murine-mammary-tumor virus (MMTV)-c-*neu*) transgenic mouse model [14,15]. HER-2/*neu*, an abbreviation for human epidermal growth factor receptor-2, is a proto-oncogene, which when activated by mutation or overexpressed has a role in uncontrolled cancer cell growth. The protein product of the Her-2/*neu *gene is overexpressed in 25 to 30% of human breast cancers. A significant proportion of human intraductal breast carcinomas (ductal carcinoma *in situ*) demonstrate Her-2/*neu *amplification/overexpression, suggesting that the functional activity of this oncogene is enhanced early in the progression of malignant breast disease. Virgin activated Her-2/*neu *transgenic mice have a 100% incidence of mammary cancer and a high multiplicity of mammary cancers [14,15]. A full-term pregnancy does not increase the incidence or multiplicity of mammary adenocarcinomas in the activated *neu *transgenic mice but does decrease the size and metastatic potential of the mammary tumors in the activated *neu *transgenic mice [16]. As these two models mimic many features of major subsets of human breast cancer, we tested whether a short-term exposure to estrogen and progesterone (*p53*-null and MMTV-*neu *models) or just estrogen (MMTV-*neu *model) would induce a protective effect on spontaneous tumorigenesis.

## Materials and methods

### Mice

BALB/c mice were bred and maintained at Baylor College of Medicine. The donor mice were BALB/c *p53 *homozygous null, and the recipient mice were *p53 *wild type. FVB (activated *neu*) mice were bred and maintained at the Cancer Research Laboratory, University of California, Berkeley, California, and at Texas Tech University of Health Sciences Center, El Paso, Texas. All mice were maintained in conventional mouse facilities with food and water provided *ad libitum*, and the room temperature was set at 70°F (21°C). The animal facilities at Baylor College of Medicine and University of California are accredited by the American Association of Laboratory Animal Care. All experiments followed NIH guidelines for th ecare and use of mice.

### *p53*-null model

The basic transplantation protocol for the experiments using the *p53*-null model was as described [10]. In brief, fragments of mammary ducts from 8 to 10-week-old female *p53*-null mice were transplanted into the cleared mammary fat pads of 3-week-old or 11-week-old female mice. Although the *p53 *deletion is the same in all donor mice, the array of secondary alterations important for neoplastic development include both common and unique events. The consequence is that the tumorigenic capabilities of mammary gland fragments vary over a small range between donor mice in the same host environment. Thus, the time to palpable tumor occurrence in 50% of animals in any two untreated groups in two different experiments may be different (for example 60 weeks versus 50 weeks). Thus, each experiment always has an untreated control group for assessment of the effect of a particular treatment. In all the transplantation experiments described here, three different donors were used for each experiment with equal representation in the different groups.

In experiment 1 there were two groups of mice. Group 1 comprised untreated control mice and group 2 comprised mice that received Silastic tubing containing 50 μg of estradiol-17β and 20 mg of progesterone for the period of weeks 2 to 4 after transplantation. The Silastic tubings were implanted subcutaneously dorsally and removed after the 2-week period. Mice were palpated weekly until 45 weeks after transplantation. At that time, the mammary fat pads free of palpable tumors were either collected for the preparation of epithelial cell pellets and frozen or prepared as whole mounts. This experiment was repeated identically and designated experiment 1A. Additionally, transplants were collected between weeks 8 and 16 after transplantation to assess proliferation activity by bromodeoxyuridine (BrdU) immunohistochemistry. The mice were injected intraperitoneally with BrdU 2 hours before tissue collection and the samples were processed as described previously [17]. A total of 500 cells were counted from each fat pad under ×10 magnification. There were four fat pads in each treatment group. In experiments 1 and 1A, a total of 66 transplants per group were assessed for tumorigenic potential.

Experiment 2 was started 12 months after experiment 1A and addressed the question of whether the developmental stage of the mammary epithelium influenced the response to the short-term hormone exposure. In this experiment there were four groups of mice. Groups 1 and 2 were identical to the groups in experiment 1. Thus, epithelial cells were actively proliferating as the ducts were filling the fat pad. The proliferation index at this stage was about 8% [11]. Group 3 comprised mice that received the hormone treatment at 23 to 25 weeks after transplantation at a period when the mammary epithelium had filled the fat pad and proliferation was in a steady state but tumors were starting to appear. We included a positive control as group 4, in which the mice were exposed to a 5 mg pellet of tamoxifen between weeks 11 and 24. Previous experiments had shown that lifetime exposure to tamoxifen prevented the development of mammary tumors in this model system [18]. The mice were palpated weekly until 50 weeks after transplantation.

Experiment 3 tested whether pretreatment of the recipient mice with hormones could provide a protective effect on mammary epithelium that had not been directly exposed to the added hormone exposure. In this experiment there were four groups of mice. Groups 1 and 2 were as in experiment 1, in which the duct fragments were transplanted into 3-week-old mice and estrogen/progesterone was provided at 2 to 4 weeks after transplantation. Groups 3 and 4 contained mice that received the hormones for 2 weeks in one group (the other group was untreated age-matched controls), followed by a rest period of 4 weeks, followed by transplantation of the duct fragments into the cleared fat pads of 11-week-old mice. The mice were palpated weekly until 60 weeks after transplantation.

Whole-mount preparations were made from four mammary glands from each of the groups in experiments 1 to 3 at 4 weeks after the removal of the Silastic tubing implants. In all experiments there was no significant difference between the treated and untreated outgrowths in the percentage of mammary fat pad filled by the implants. All palpable tumors were fixed in 4% paraformaldehyde for 2 to 4 hours, embedded in paraffin and processed for staining with hematoxylin/eosin. As the transplanted mammary epithelium develops primarily mammary adenocarcinomas but also hemangiosarcomas (at 10% incidence, presumably because of endothelium that is also transplanted as part of the duct fragment), we assessed all tumors histologically to confirm the cell type of origin. In the text throughout, tumor refers to mammary adenocarcinoma. Mammary adenocarcinoma incidences were evaluated statistically with Fisher's exact test. The BrdU immunohistochemistry was performed as described [11].

### Activated MMTV-*neu *model

The design of the experiments using the MMTV-*neu *model was slightly different. FVB transgenic mice were treated for 3 weeks, starting at 7 weeks of age, with 100 μg of estradiol in Silastic capsules. Another set of transgenic animals were treated with 100 μg of estradiol and 15 mg of progesterone, also in Silastic capsules. The doses of estradiol used result in pregnancy levels of estradiol in the circulation [19]. The control animals received empty Silastic capsules for the same duration. Mice were palpated once every week for 8 months to monitor for mammary cancer development. Histopathological examination was performed to confirm the carcinomatous nature of the palpable tumors. Mammary cancer incidence was evaluated statistically with the χ^2 ^test.

### Hormone preparations

All mice received either empty Silastic tubing or the hormones in Silastic tubing. For both the *p53*-null model and the *neu *model, the tubings were prepared with the same protocol. The hormones were packed in individual Silastic capsules (Dow Corning; size 0.078 inch (2 mm) internal diameter, 0.125 inch (3.2 mm) outside diameter, 2 cm in length). Estradiol-17β (Sigma, St Louis, MO, USA) was packed in the Silastic capsules in a cellulose matrix. Progesterone (15 mg; Sigma) was packed into the Silastic capsules also in a cellulose matrix. All these capsules were primed by soaking overnight in medium 199 (Gibco, Grand Island, NY, USA) at 37°C. Silastic capsules were implanted subcutaneously dorsally.

### RNA analysis

A group (*n *= 3) of control animals and animals treated with estradiol or estradiol plus progesterone were killed immediately after the 3 weeks of hormone treatments. Mammary glands were removed from all the groups for the transgene expression analysis. Another set of animals (*n *= 15) received the above-mentioned hormone treatments and were killed at 9 months of age. Mammary tumor and mammary tissue adjacent to the mammary tumors were excised, snap-frozen in liquid nitrogen, and stored at -80°C for molecular analysis. Total RNA isolated from the normal mammary gland, mammary tumor, and mammary tissue adjacent to the mammary tumor was subjected to real-time RT-PCR analyses to study the expression of the transgene [20]. RNA was isolated with Trizol reagent, and real-time reverse transcription was performed with the one-step QuantiTect SYBR Green kit (Qiagen Inc., Valencia, CA, USA) in accordance with the manufacturer's specifications. 18S RNA was used as standard and positive control in these assays. Fold changes between samples for relative Her-2/*neu *transgene mRNA expression were calculated from the differences in Δ*Ct *values between the two samples (ΔΔ*Ct*) and the equation Fold change = 2^-ΔΔ*Ct*^. Real-time RT-PCR was performed with the oligonucleotide primers 5'-GGAACCTTACTTCTGTGGTGT-3' and 5'-GGAAAGTCCTTGGGGTCTTCT-3' targeting the SV40 poly(A) region of the transgene. Cyclin D1 expression was performed on the mammary tumors from the control and hormone-treated groups with the oligonucleotide primers 5'-CATCAAGTGTGACCCGGACTG-3' and 5'-CCTCCTCCTCAGTGGCCTTG-3'.

## Results

### Effect of short-term hormone stimulation on *p53*-null mammary tumorigenesis

**Figure 1:**
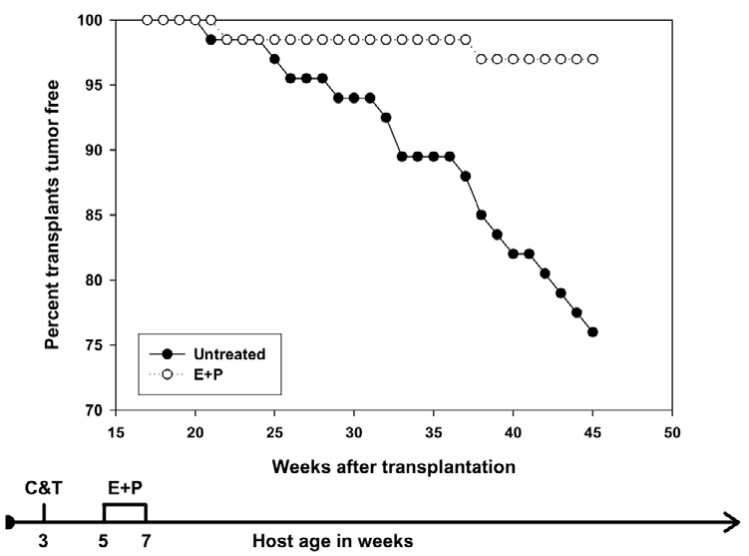
Effect of short-term hormone treatment on tumorigenesis in *p53*-null mammary epithelial transplants. The data are the sum of two identical experiments (experiments 1 and 1A). Mice treated with estradiol and progesterone had a significant decrease in tumor incidence (*p *< 0.05). Filled circles, untreated; open circles, hormone-treated. The flow diagram illustrates the experimental treatment plan. C, clear mammary gland; T, transplant; E, estrogen; P, progesterone.

The tumorigenic response of the *p53*-null mammary epithelium exposed to estrogen and progesterone combination at 2 to 4 weeks after transplantation is shown in Figure 1 and Table 1. In the control transplants, 16/66 (24%) produced tumors by 45 weeks after transplantation, with an initial tumor latency of 20 weeks. This incidence is consistent with previous studies [10,18]. In the hormone-treated mice, only 2/66 (3%) produced tumors (*p *< 0.05). This experiment demonstrates conclusively that a short-term hormone treatment can delay tumor development in a non-carcinogen model of mammary cancer.

**Figure 2:**
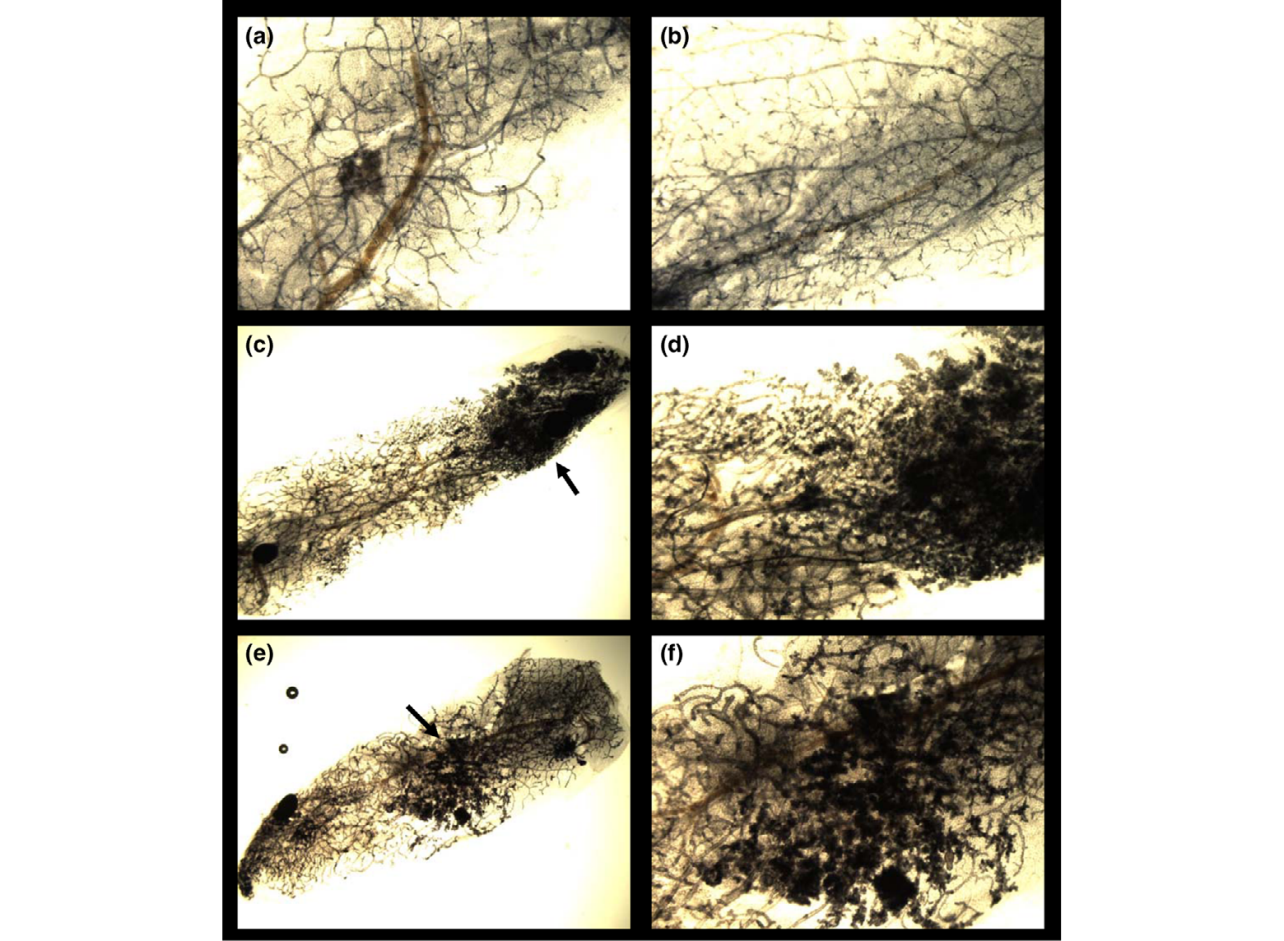
Effect of short-term hormone treatment on gland morphology in *p53*-null transplants. **(a,b) **Mammary whole mounts were prepared 4 weeks after the removal of hormones in mice from experiment 1A. The ductal organization at the histological and microscopic (hematoxylin/eosin-stained sections, not shown) was similar in the untreated (a) and treated (b) mice (original magnification ×2). **(c–f) **Whole mounts were prepared at 45 weeks after transplantation in transplants that had not yet developed palpable tumors. A large number of transplants in the untreated group (46%) (original magnifications ×1 (c) and ×2 (d)) and in the treated group (42%) (original magnifications ×1 (e) and ×2 (f)) contained areas of ductal hyperplasias (arrows).

Whole-mount analysis of the glands in experiments 1 and 1A at 4 weeks and at 8 to 12 weeks after transplantation did not indicate a significant difference in outgrowth morphology. The outgrowths exhibited normal alveolar differentiation to 2 weeks of estrogen/progesterone exposure at 4 weeks after transplantation. At 8 to 12 weeks after transplantation, the outgrowths appeared as normal mammary duct arborization with no evidence of ductal hyperplasia (Figure 2). However, whole-mount analysis of the glands at 45 weeks after transplantation revealed extensive ductal hyperplasia in both control and hormone-treated glands. Premalignant lesions were present in 13/31 glands in the estrogen/progesterone-treated group (42%) and 6/13 glands in the untreated controls (46%) (Figure 2; *p *> 0.05). These data suggest that hormones act to block premalignant progression and not the onset of hyperplastic growth.

### Effect of developmental state on tumorigenesis in *p53*-null mammary epithelium

**Figure 3:**
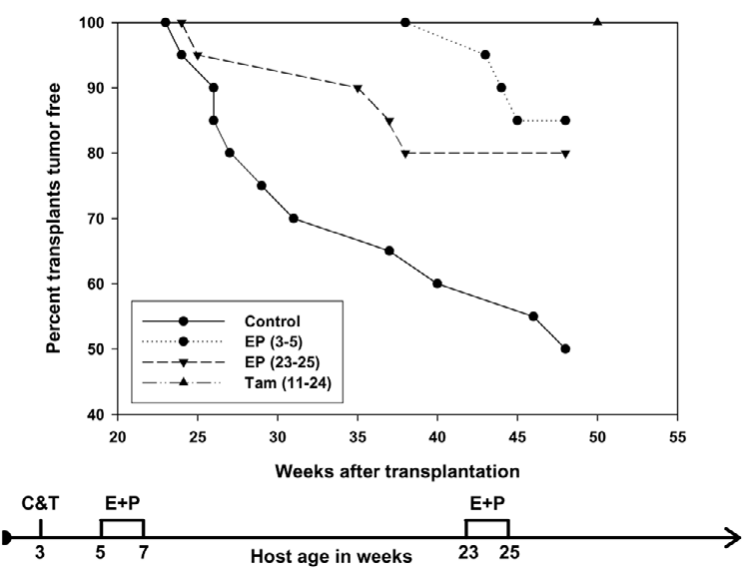
The effect of duration of hormone exposure on tumorigenesis in *p53*-null mammary epithelial transplants. The effect of hormone exposure was compared with that of a hormone receptor antagonist, tamoxifen. Mice treated with estradiol and progesterone, either early or late, or treated with tamoxifen, had a significant decrease in tumor incidence (*p *< 0.05). Filled circles and solid line, control; filled circles and dotted line, early hormone exposure; inverted triangles and broken line, late hormone exposure; upright triangle, tamoxifen. The flow diagram illustrates the experimental treatment plan.

The tumorigenic response of the mammary epithelium exposed to estrogen and progesterone combination at 23 to 25 weeks after transplantation is shown in Figure 3 and Table 1. In the control transplants, 10/20 (50%) produced tumors by 48 weeks after transplantation, with an initial tumor latency of 24 weeks. In the transplants exposed to hormone combination when they were actively filling the fat pad, the tumor incidence was 3/20 (15%), with an initial tumor latency of 44 weeks. In contrast, the transplants exposed to the hormone combination much later after transplantation had an initial tumor latency equivalent to the controls, but the final tumor incidence (4/20; 20%) was not significantly different (*p *> 0.05) from that in the group exposed to hormones early after transplantation. The transplants exposed to tamoxifen for only 3 months did not develop any tumors after 50 weeks. This experiment demonstrates that a short-term treatment administered at either the actively proliferating or the steady-state stage of mammary development can delay tumorigenesis.

### Effect of hormone pretreatment of the host on tumorigenesis in *p53*-null mammary epithelium

**Figure 4:**
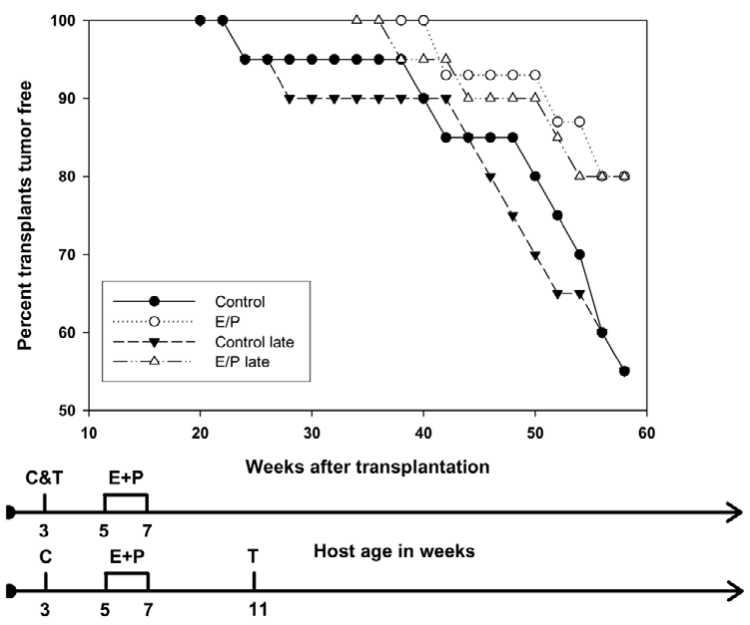
The effect of hormone-induced systemic changes on tumorigenesis in *p53*-null mammary epithelial transplants. Mammary epithelial transplants in a host exposed to estradiol and progesterone before transplantation (open triangles and dot-dashed line) also had a significant decrease in tumor incidence (*p *< 0.05) compared with mice not exposed to the hormones (filled triangles and broken line). The flow diagrams illustrate the experimental treatment plans.

The tumorigenic capability of the *p53*-null mammary epithelial cells either directly exposed to estrogen/progesterone or just to the estrogen/progesterone-treated host is shown in Figure 4 and Table 1. The tumorigenic capability was the same whether the target epithelial cells were directly exposed to estrogen/progesterone or transplanted into an estrogen/progesterone-treated host environment (3/15 versus 4/20, respectively; *p *> 0.05). The tumorigenic response was decreased from 45% (18/40 combined controls) to 20% (7/35 combined treatment; *p *< 0.05).

**Figure 5:**
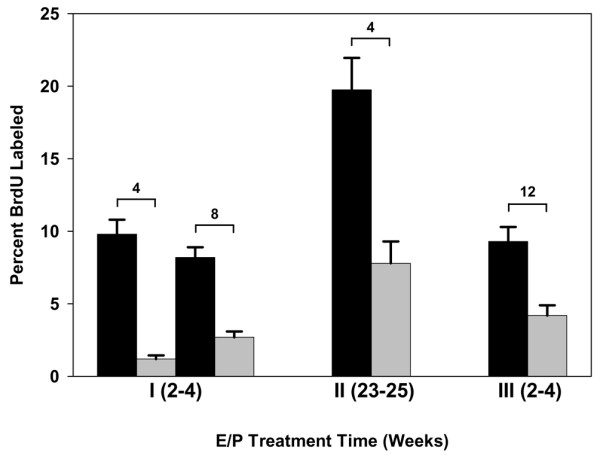
Bromodeoxyuridine-labeling index (number labeled per 500 cells) in hormone-treated *p53*-null mammary epithelial transplants. Transplants from each experiment (1, 2 and 3) were assayed for bromodeoxyuridine. The black bars represent untreated transplants, the gray bars hormone-treated transplants. The number above each pair of bars indicates the number of weeks after the removal of hormones. In experiment 3, 12 weeks after the removal of hormone represents 8 weeks after transplantation. There were four transplants per treatment group. Five hundred cells were counted in each transplant. All four comparisons were significantly different (*p *< 0.05).

**Figure 6:**
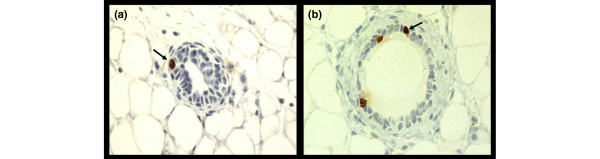
Immunohistochemistry of bromodeoxyuridine-labeled mammary epithelial cells. The brown nuclei (arrows) represent the uptake of bromodeoxyuridine into cells undergoing DNA synthesis. **(a) **Untreated; (**b**) treated with estrogen plus progesterone.

The proliferative state of the mammary epithelial cells was assessed at 4 to 12 weeks after removal of the hormones in all three experiments (Figures 5 and 6). In experiment 1, at 4 and 8 weeks after hormone removal, the control transplants showed a BrdU-labeling index of 9.8 and 8.2, respectively, in comparison with 1.5 and 1.8, respectively, in the hormone-treated transplants (*p *< 0.05). In experiment 2, at 4 weeks after hormone removal (that is, 26 weeks after transplantation), the control transplants showed a BrdU-labeling index of 19.8 in comparison with 7.8 in the hormone-treated transplants (*p *< 0.05). In experiment 3, at 12 weeks after hormone removal (that is, 8 weeks after transplantation into 11-week-old mice), the control transplants had a BrdU-labeling index of 9.5 in comparison with 4.5 in the transplants in the hormone-treated mice (*p *< 0.05).

### Effect of hormone treatment on MMTV-*neu *mammary tumorigenesis

**Figure 7:**
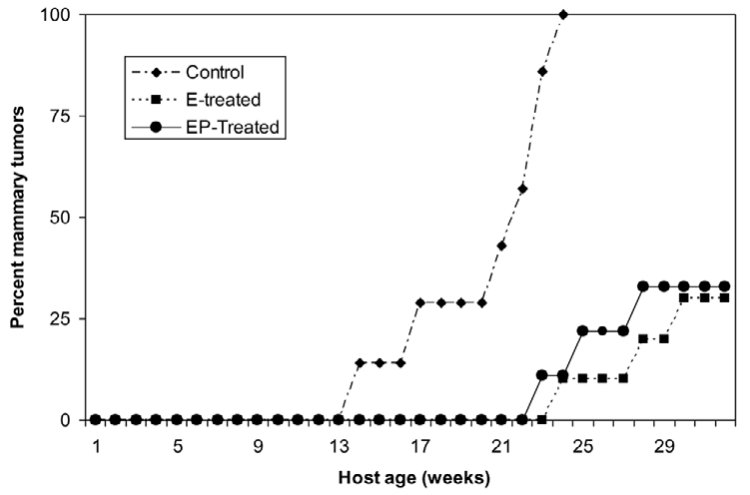
Effect of short-term hormone treatment on mammary carcinogenesis in the activated Her-2/*neu *transgenic mice. Mice treated with estradiol alone (7/21) or estradiol plus progesterone (9/24) had a significant decrease in the incidence of mammary tumors compared with the controls (20/20) at 8 to 9 months of age (*p *< 0.01).

The marked response of the *p53*-null mammary epithelium to a short-term exposure to hormone raised the question of whether a similar response would occur in a more tumorigenic model of mammary tumorigenesis. The MMTV-activated *neu *model provided such a model, because tumors develop rapidly and with high multiplicity. The effect of either short-term treatment with estradiol or estradiol plus progesterone on mammary carcinogenesis was tested. The data in Figure 7 and Table 1 indicate that both treatments were equally effective in providing protection. Mammary cancer incidence after treatment with estradiol alone (33%) or with estradiol plus progesterone (37%) was significantly decreased in comparison with the controls (100%).

**Figure 8:**
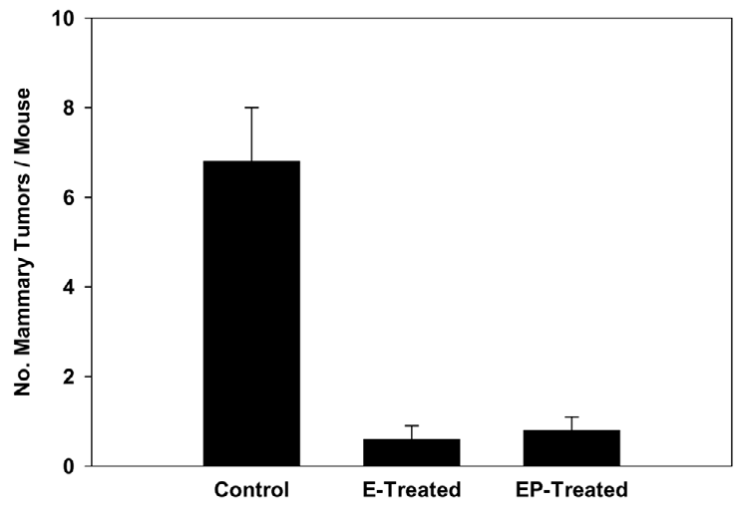
Effect of short-term hormone treatment on mammary cancer multiplicity in the activated Her-2/*neu *transgenic mice. The group of mice (*n *= 21) treated with estradiol alone developed a total of 13 mammary tumors and the group of mice (*n *= 24) treated with estradiol plus progesterone developed a total of 20 mammary tumors by 8 to 9 months of age; in comparison, the group of control mice (*n *= 20) developed a total of 136 mammary tumors (*p *< 0.005). Results are expressed as means ± SEM.

**Figure 9:**
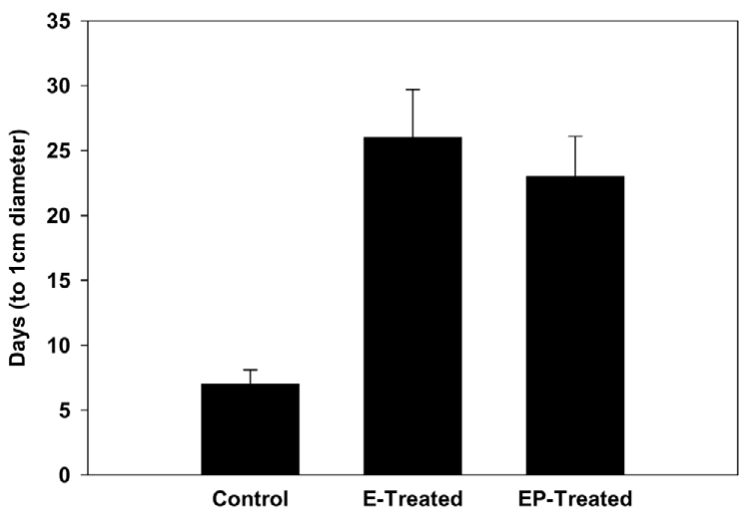
Effect of short-term hormone treatment on tumor growth rate in the activated Her-2/*neu *transgenic mice. Treatment with estradiol alone or estradiol plus progesterone significantly increased the time taken for the mammary tumors to grow from initial palpation to a diameter of 1 cm in comparison with the controls. Results are expressed as means ± SEM.

All the control mice developed mammary cancers by 5 months of host age with a multiplicity of 6.8 cancers per mouse. Treatment with estradiol (0.6 cancers per mouse) or estradiol plus progesterone (0.8 cancers per mouse) drastically reduced mammary cancer multiplicity and also approximately doubled the mammary cancer latency (Figures 8 and 9). This experiment was repeated once with identical results. A second repeat experiment was not informative because the control untreated mice did not develop tumors with their usual early latency of 13 to 15 weeks of host age but started to develop tumors only at 30 weeks.

Mice were examined twice weekly beginning after the hormone treatments, and the earliest tumors were detectable by palpation (Figure 9). The sizes of tumors were recorded after each examination. In control mice the average age at which palpable tumors were first detected was 95 days. Tumors were initially palpated 142 days in estradiol-treated mice and at 138 days in mice treated with estradiol plus progesterone. Tumor growth was monitored until the tumor in each mouse had reached a diameter of 1 cm. The difference in tumor growth rate between the control and hormone-treated mice was significant. There was almost a threefold difference in the time required for the mammary tumors to grow to 1 cm in diameter (after initial palpation) in mice treated with estradiol or estradiol plus progesterone compared with the controls.

**Figure 10:**
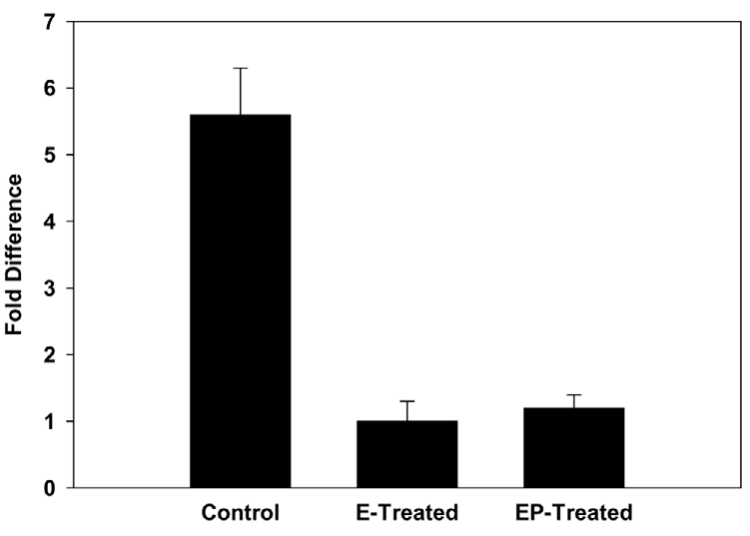
Cyclin D1 mRNA expression levels in mammary tumors arising in activated Her-2/*neu *mice. There was an approximately sixfold increase in the level of cyclin D1 in mammary tumors arising in control mice in comparison with tumors arising in hormone-treated mice. Results are expressed as means ± SEM.

Real-time PCR analyses demonstrated that expression of the transgene was not significantly altered by the transgene in the normal mammary tissue, mammary tumors and the mammary tissue adjacent to the mammary tumor. There was a significant difference in the expression levels of cyclin D1 (Figure 10). Mammary tumors from the control group had a significantly higher (about 5.6-fold) expression of cyclin D1 in comparison with animals treated with estradiol alone or estradiol plus progesterone.

## Discussion

The experiments reported here are the first to address the question of whether short-term hormone treatment can delay tumorigenesis in genetically engineered models of mammary cancer. All previous experiments, with one exception in which radiation was used, were performed in rodent models treated with chemical carcinogen. The results show clearly that a short-term hormone treatment of estrogen with or without progesterone can significantly delay tumorigenesis in two different genetically engineered mouse models. The models differ in fundamental mechanisms of mammary tumorigenesis. The *p53*-null epithelium is a model in which a major tumor suppressor gene is deleted and aneuploidy is a major feature of the mammary tumors. The tumors arise over a 14-month period and the incidence reaches only 50 to 60% during this period. In contrast, the activated-*neu *model represents the overexpression of an oncogene, and tumors arise very rapidly and with high multiplicity. These results need to be repeated with other genetically engineered mouse models, such as the *BRCA1 *and c-*myc *models, to determine the wider applicability of this effect of hormones. Conceptually, this result is important because the genetically engineered models replicate more faithfully basic features of human breast cancer than do the chemical carcinogen models.

Several results are of general interest. In the *p53*-null model, the mature gland as well as the developing (that is, immediately post-pubescent) gland was responsive to the protective state induced by the hormone treatment. This result implies that there is no unique developmental state of susceptibility. In the MMTV-activated *neu *model, the effect of hormones was tested on the developing mammary gland at 7 to 10 weeks of age. These results suggest that the translation of this approach to humans is not limited to the young post-pubescent female but can be applied to the young to middle-aged adult female. Although the emphasis from the human epidemiology studies has always been on early first pregnancy as a critical determinant for protection, the use of a specific hormone combination for short durations might be applicable to a wider age range than previously thought. This conclusion is in line with experiments in the rat model that show a protective effect of hormones even after initiation by a chemical carcinogen. Huggins and colleagues originally reported that estradiol and progesterone given for 30 days, beginning 15 days after carcinogen administration, inhibited the appearance of mammary cancers in rats treated with chemical carcinogens [4]. We also demonstrated that protection against mammary carcinogenesis could be achieved by treatment with physiological levels of estradiol and progesterone for 21 days or less [21,22]. In the MMTV-activated *neu *model, 100 μg of estradiol in the Silastic tubing yielded a circulating level of serum estradiol of 98.56 ± 8.37 pg/ml (SEM) (*n *= 6) at 21 days after implantation of the tubing. The groups treated with estradiol plus progesterone yielded similar results (L Rajkumar, unpublished data).

In support of the idea that the mature gland is responsive to the protective effects of a short-term exposure to estrogen and progesterone is the observation that this hormone combination seems to be acting to delay premalignant progression. The presence of frequent hyperplasia in the hormone-treated gland but the absence of invasive cancers supports this conclusion. There is some limited information in the literature that supports this idea. Reddi and colleagues have presented data that show the presence of microtumors in the glands of hormone-treated rats under conditions in which the controls had a high incidence of invasive cancers [23]. The data presented in the present study would be the first demonstration of this result in mice. Experiments that test the growth potential of these microtumors or hyperplasias by transplantation into control animals have yet to be reported. It is evident that there is a point in premalignant progression at which the cells are no longer susceptible to the preventive effects of this hormone combination. Examination of the tumor incidence curves of mice that were exposed to hormones at 23 to 25 weeks of age clearly show that the first tumors appeared with a latency similar to that in control mice. Thereafter, this group behaved similarly to the mice that received the early exposure to hormone.

One of the cellular mechanisms underlying the protective effect involves a diminution of the proliferative potential of the mammary cells. All experiments in the *p53*-null model demonstrated that the proliferative index of the hormone-treated cells was reduced by 53% to 85% of that of untreated control mammary cells at the steady-state level observed in the mature gland. Interestingly, the ability of the short-term hormone treatment to stimulate proliferation and differentiation during the expansion period at 2 to 6 weeks after transplantation was not compromised, because the extent of filling of the fat pad was the same in the two groups at 6 weeks after transplantation. This suggests that the mechanism for controlling proliferation in the two states (that is, expanding versus a steady-state cell population) might be different either at the level of the cell type that is proliferating in the two states or at the molecular level. We have not yet evaluated the proliferative indices of the hyperplasias in the control and hormone-treated mice because the original observations that determined the presence of these hyperplasias in the hormone-treated mice were performed on whole mounts of the glands, and, indeed, the result was surprising to us. However, in the MMTV-activated *neu *model, the decrease in proliferative activity was also observed in the tumors arising in the hormone-treated mice. This decrease was manifested at the cellular level in a decrease in cyclin D1 expression.

Perhaps the most surprising result is the apparent systemic effect of the hormones. This experiment demonstrated conclusively that the effect of the hormones can be mediated, in part, by changes induced at the systemic level and/or the mammary stroma. This idea was presented by Thordarson and colleagues in studies on the carcinogen-induced rat mammary system [24]. Attempts to test this hypothesis were only partly successful [25]. The results presented here demonstrate conclusively that hormone-induced effects at the systemic level and/or at the mammary stroma can affect tumorigenesis in the *p53*-null mammary cells. Such an effect is not without precedent. Barcellos-Hoff and Ravani demonstrated that irradiated stroma can alter premalignant progression of a mouse mammary outgrowth line, COMMA-D [26]. Maffini and colleagues showed that stroma treated with a chemical carcinogen (*N*-methyl-*N*-nitrosourea) can enhance the progression of rat mammary cells to mammary tumors [27]. Schedin and colleagues [28] demonstrated that the extracellular matrix of mammary stroma from different reproductive states alters mammary epithelial morphogenesis as well as mammary epithelial growth rate. Specifically, they showed that matrix isolated from parous stroma delayed glandular morphogenesis. The cellular and molecular mechanisms underlying the changes systemically or at the mammary stroma are beginning to be identified. Alterations in both transforming growth factor-β signaling [26] and growth hormone signaling [23,24] have been implicated. Interestingly, we could not demonstrate altered systemic insulin-like growth factor-1 levels in our hormone-treated mice at 4 weeks after hormone removal (D Medina and A Lee, unpublished data).

Finally, it is apparent that the preventive activity can be induced by a modest dose of estrogen alone (100 μg) or a combined dose of estrogen and progesterone. Previously we had shown that short-term sustained exposure to 100 μg of estradiol resulted in pregnancy levels of estradiol in circulation [19,29]. We further showed that a pregnancy level of estradiol alone or a combination of estradiol and progesterone was highly effective in inducing refractoriness to chemical carcinogen-induced rat mammary carcinogenesis. Similarly to the protection observed in rats by a pregnancy level of estradiol, activated Her-2/*neu *mice are also rendered refractory to mammary cancer development by short-term hormone treatments.

These studies illustrate two of the major paradoxes of the role of hormones in mammary tumorigenesis. On the one hand, a short duration of estrogen and progesterone or estrogen alone imparts a protective effect on tumor development. On the other hand, continuing the same dose of hormones for a prolonged period strongly stimulates the development of tumors in this same system as well as in other mouse models [5,18,30]. This same result has been reported in the rat mammary tumor system [31]. Different mechanisms underlying the protective effects have been proposed by several investigators. Sivaraman and colleagues and Ginger and colleagues [21,32,33] emphasize the induction of a different developmental fate as a consequence of hormone exposure. Thordarson and colleagues have argued for a systemic effect involving the downregulation of pituitary hormones [24,34,35]. In either event, one would have to conclude that continuing exposure to hormones overrides these mechanisms. Mechanistically, the basis for this override is not clear.

The second paradox is that by either exposing the mammary gland to a short duration of hormones or blocking the same hormone pathway (for example by exposure to tamoxifen) a similar result is generated, namely a decrease in tumorigenic potential. However, the cellular pathways perturbed by the two treatments might be entirely different because tamoxifen-treated outgrowths do not show the presence of hyperplasias that occur in the hormone-treated outgrowths.

In summary, these studies provide a further rationale for considering the use of short-term hormone exposure as a preventive modality, particularly in high-risk individuals. Despite the extensive documentation of the preventive potential of early full-term pregnancy and its mimicry by estrogen and progesterone, there is great resistance to the use of these hormones as a preventive modality. In part, the resistance is due to the overwhelming data showing that prolonged exposure to estradiol and progesterone increases the risk for breast cancer [36]. This resistance might be mitigated by recent data indicating that hormone replacement therapy with estrogen alone does not increase the risk for breast cancer [37]. Perhaps this resistance will be overcome once the mechanisms underlying the preventive effects of specific hormone combinations and duration of exposure are understood.

## Conclusion

These studies demonstrate that short doses of the hormones estrogen and progesterone induce a long-lasting protective effect on mammary tumorigenesis in two genetically engineered mouse models. At least part of the effects of the hormone treatment is mediated through systemic and/or mammary stroma alterations.

## Abbreviations

MMTV = murine-mammary-tumor virus; PCR = PCR; RT = reverse transcriptase.

## Competing interests

The authors declare they have no competing interests.

## Authors' contributions

Each author contributed equally to the results reported in this manuscript. All authors read and approved the final manuscript.

## Figures and Tables

**Table 1:** Tumorigenesis in short-term hormone-treated mice

Experiment	Group	Tumors/transplants (percentage)	Percentage inhibition	Experiment duration (weeks)
1/1A	Untreated	16/66 (24)	-	45
	E/P (5–7)	2/66 (03)^a^	87.5	45
2	Untreated	10/20 (50)	-	48
	E/P (5–7)	3/20 (15)^a^	70	48
	E/P (23–25)	4/20 (20)^a^	60	48
	Tamoxifen (11–24)	0/20 (0)^a^	100	48
3	Untreated, trans. (3)	9/20 (45)	-	58
	E/P (5–7), trans. (3)	3/15 (20)^a^	56	58
	Untreated, trans. (11)	9/20 (45)	-	58
	E+P (5–7), trans. (11)	4/20 (20)^a^	56	58
4	Untreated	15/15^b ^(100)		24
	E/P	6/15^b ^(37) ^a^	63	32
	E	5/15^b ^(33) ^a^	67	32

E, estrogen; P, progesterone; trans., transplantation. ^a^*p *< 0.05; ^b^in this experiment, the column refers to the number of tumor-bearing mice/total number of mice.
